# Addressing COVID-19 vaccine hesitancy: Lessons from the role of community participation in previous vaccination programs

**DOI:** 10.34172/hpp.2021.54

**Published:** 2021-12-19

**Authors:** Aanuoluwapo Adeyimika Afolabi, Olayinka Stephen Ilesanmi

**Affiliations:** ^1^Department of Community Medicine, College of Medicine, University of Ibadan, Ibadan, Oyo State, Nigeria; ^2^Department of Community Medicine, University College Hospital, Ibadan, Oyo State, Nigeria

**Keywords:** Community involvement, COVID-19, COVID-19 vaccines, Vaccination, Vaccination refusal

## Abstract

The acceptance of the coronavirus disease 2019 (COVID-19) vaccine has been described as a gateway to attaining herd immunity against severe acute respiratory syndrome coronavirus 2 (SARS-CoV-2). The critical role of community participation (CP) has been successfully demonstrated in previous vaccination programs globally. This perspective therefore aimed to describe how CP could be used to promote COVID-19 vaccination acceptance. To promote COVID-19 vaccine acceptance, it is required that a mapping of community assets, resources, civil-based organizations, and stakeholders is done to gain insight into the community culture and value in relation to COVID-19 vaccine. This will help to address the misconceptions while prompting COVID-19 vaccination sensitization activities that are relevant to each community. It is required that policy makers understand that the adoption of a comprehensive grassroots approach lends a voice to the community and helps to utilize community-initiated and community-driven ideas on promoting COVID-19 vaccine acceptance.

## Introduction

The coronavirus disease 2019 (COVID-19) pandemic has ushered in a global public health threat hitting both developing and developed countries hard.^1,2^ The lack of COVID-19 vaccines contributed to an increase in COVID-19 cases and deaths, with more than 500 000 deaths recorded globally particularly among people with underlying health conditions, such as diabetes and immune-compromised health status. Few months ago, some COVID-19 vaccines were rolled out after approval from global vaccine regulatory bodies.^3^ The production of effective COVID-19 vaccines therefore indicated an effective stride towards halting the spread of severe acute respiratory syndrome coronavirus 2 (SARS-CoV-2), thereby resulting in a significant decline in COVID-19 cases and deaths. Lack of confidence in the COVID-19 vaccines have been reported.^4^ Many individuals have reported many side effects, such as blood clots, fever, body weakness, and malarial symptoms following the acceptance of the first and/or second dose of any of the COVID-19 vaccines.^5,6^ These reports have therefore promoted COVID-19 vaccine hesitancy in community settings. Many other factors have been implicated in COVID-19 vaccine hesitancy. These include (a) Psychological and contextual variables, which describe a unit increase in the odds of vaccine hesitancy following an increase in the perceived risks of COVID-19 vaccine; (b) Higher belief in the superiority of natural immunity increased the odds of vaccine hesitancy by nearly three times; and (c) Distrust in COVID-19 mitigation measures increased the odds of vaccine hesitancy.^7-9^

Community participation (CP) has been described as a powerful vehicle for producing environmental and behavioral changes that will improve the health of the community and its members.^10^ Vaccine hesitancy has been described as the delayed acceptance or blunt refusal of vaccines amidst a global health threat. Community resistance and diplomatic friction have been reported. For instance, the lack of strong communication strategies tailored for the grassroots accounted for a drastic decline in polio vaccine uptake in Nigeria in 2003.^11^ Non-acceptance of the oral polio vaccine resulted from a rumor on the inclusion of an anti-fertility agent into the vaccine as a method of placing a check on population growth. To address this growing public health challenge, community stakeholders and members were mobilized. To further promote polio vaccination uptake, the United Nations Children’s Fund (UNICEF) implemented several interventions.^12^ These included community awareness sessions, mobilization of local and religious leaders, outreach activities through health workers, and the establishment of community volunteer networks. To promote COVID-19 vaccine acceptance, it is required that a mapping of community assets is immediately undertaken. Asset mapping is a community development strategy that helps to identify and document existing resources, including institutions, local leadership, and economic resources.^12^ Asset mapping is needed to gain insight into the level of vulnerability of each community to COVID-19, level of trust in recommended public health safety measures, level of vaccine acceptance, and factors that influence vaccine hesitancy among members of the population. CP has been documented in improving community hygiene practices in the prevention of *Lassa*fever and other infectious diseases.^13^ However, paucity of knowledge yet exists on the mechanism behind CP in the context of the COVID-19 vaccine. Given that acceptance of the COVID-19 vaccine is a major requirement towards attaining herd immunity against SARS-CoV-2,^14^ this perspective aimed to describe how CP could be adopted to promote COVID-19 vaccination acceptance.

## Mechanisms used for community participation in previous vaccination programs

### HIV/AIDS vaccine demonstration in Nigeria

A community stakeholder engagement activity during the vaccine demonstration project undertaken by the Nigerian Canadian Collaboration on AIDS vaccine (NICCAV) in Jos, Nigeria exemplifies evidence-based mechanism for CP.^15^These are discussed as follows:

Through the insight gained into the community’s exiting partnerships, programs, priorities, and resources, NICCAV was able to identify 65 stakeholders involved in the HIV response in Jos, Plateau State. These included policy makers from the Ministry of Health and the National Agency for the Control of AIDS, representatives of civil-based organizations, journalists, research team members, members of groups living with HIV, as well as leaders of opinion groups and religious organizations. Each community-based organization was provided with support on integrating HIV/AIDS awareness into public education. To commemorate the HIV vaccine awareness day and world AIDS day, joint research literacy activities were conducted. In the same vein, HIV counselling and testing activities were introduced to augment HIV awareness programs undertaken by community-based organizations.^15^

Regular meetings and feedback sessions were held monthly, thus providing an opportunity to identify challenges and chart possible solutions. A community advisory board made up of an 11-man committee comprising of representatives of opinion groups, media organizations, traditional rulers, and health agencies was formed. To increase public awareness about the project, a media engagement program was developed. A structured capacity-building program held once in three months was organized for 35 civil society organizations, 11 members of the community advisory board, and 14 journalists. An advocacy agenda was developed through the expertise of journalists, HIV research programmers, drug regulatory officers, and community advocates. Monitoring and evaluation of the project was done to track the progress level, and to assess if the overall aim of the project was achieved.^15^

### Promotion of routine immunization program through the post-Ebola outbreak response in Liberia 

The Ministry of Health, Liberia, and the Health Promotion unit of the United Nations Children’s Fund rolled out the social mobilization activities through the county mobilization and district mobilization coordinators.^16^ An integrated stream of activities including mobilization of town criers, mass media, religious leaders, opinion group leaders, community-based dialogues, and rapid polls through a free short message service (U-report) was organized to promote feedback during and after the Ebola outbreak response in Liberia. Overall, 229 031 homes were visited, while 5840 religious leaders and 5992 community leaders were mobilized. Nearly 3000 community meetings were held, alongside the distribution and/or production of 35 000 flyers, 5678 posters, and 60 banners. Three radio dramas were produced, and these were aired across 67 radio stations.^16^

Through these CP activities, 693 622 children received the oral polio vaccine, and 596 545 (99%) children received the measles vaccine compared to the huge decline that was recorded during the Ebola disease outbreak.^16^

## Implementation of the social mobilization network to improve routine immunization program in India

To strengthen CP to achieve the targets of the routine immunization program, India implemented the social mobilization network.^11^ Key strategies included evidence-based planning on strong outreach and advocacy, and accountability for action. The success of the social mobilization network was built on strong partnership with community stakeholders and community-based organizations. With the support of the social mobilization network, vaccine hesitancy has been sustained at less than 1%, and nearly 100% coverage of oral polio vaccine in social mobilization network areas in Uttar Pradesh and Bihar.^11^

## Theory of Change

**Figure 1:**
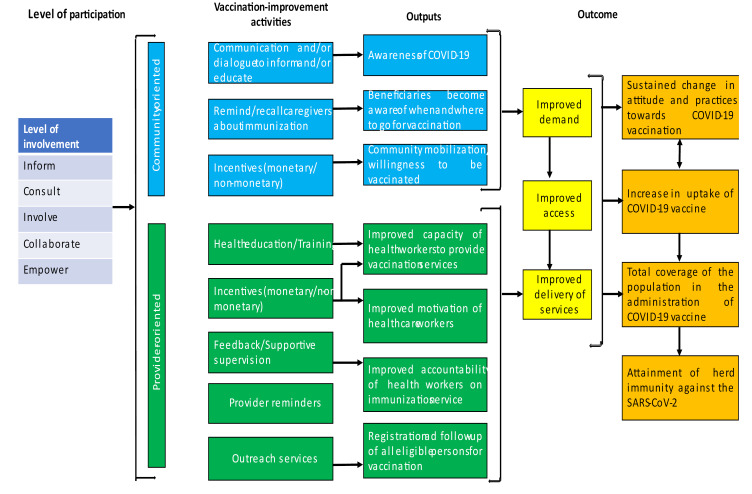
Theory of change to improve willingness to take the COVID-19 vaccine. Adapted from Jain et al.^18^

To improve the acceptance of the COVID-19 vaccine in many communities, the theory of change would be effective in driving CP.^17^The spectrum of CP involves five levels of engagement. These include: (i) Inform: A process whereby the community is reached out to as a part of the COVID-19 vaccine distribution intervention; (ii) Consult: Consulting the community to develop the vaccine distribution strategy; (iii) Involve: Involvement of the community to ensure that concerns related to their ethical issues and beliefs regarding the COVID-19 vaccine are addressed during the decision-making process. Others include: (iv) Collaborate: Collaborating with the community to develop partnerships; and (v) Empower: Empowering the community to make decisions as well as to implement and manage the COVID-19 vaccine distribution and sensitization intervention (Figure 1).^17,18^

## Way forward in addressing COVID-19 vaccine hesitancy

Communities are not a homogenous entity, but composed of individuals with different values, expectations, and different levels of health literacy.^19^ A top-down one-size-fits-all approach is therefore unrealistic in the context of the COVID-19 vaccine in promoting global health. Drawing on the lessons learnt from the effectiveness of CP in previous immunization programs, we hereby recommend the following evidence-based strategies to reduce COVID-19 vaccine hesitancy.

COVID-19 vaccination programs should adopt a community-centered approach.^20^ This implies the need for the integration of COVID-19 vaccination programs into routine healthcare programs. Evidence from the integrated management of childhood illnesses shows that the integration of vaccination programs via community values and norms reduces vaccine hesitancy.^21^ Therefore, it is required that policy makers understand that the adoption of comprehensive grassroots approach lends a voice to the community and helps to utilize community-initiated and community-driven ideas on promoting COVID-19 vaccine acceptance. In addition, the adoption of CP will help map local concerns to co-design programs with community stakeholders to reduce COVID-19 vaccine hesitancy.

Since COVID-19 vaccine hesitancy has been primarily linked to lack of confidence in the health authorities, opportunities for promoting confidence of the community members in the health authorities need to be developed.^4^ The national health authority through her local agencies should leverage and support community members that influence decision making processes. These would include teachers, heads of civil-based organizations, and opinion group leaders. Due to their engagement, members of the public would gain insight into innovations for the local context, while building their confidence in health authorities on the safety of the COVID-19 vaccine. CP is a cost-effective measure that adopts a participatory modality in improving COVID-19 vaccine uptake, thereby drastically reducing healthcare expenditure.

## Conclusion

There is a growing recognition of the critical role played by CP in the uptake of the COVID-19 vaccine as evidenced by its contributions in previous vaccination programs. CP is an essential ingredient that could reduce COVID-19 vaccine hesitancy. To address COVID-19 hesitancy and improve vaccine acceptance, the engagement of community stakeholders such as community-based organizations, leaders of opinion groups and religious organizations, media teams, as well as community members themselves should be immediately undertaken. To overcome COVID-19 vaccine hesitancy, community members should be involved in the design and implementation of the COVID-19 vaccine distribution plan. It is anticipated that if effectively implemented, adequate CP would help to improve COVID-19 vaccine acceptance and address COVID-19 vaccine hesitancy globally.

## Funding

This research was fully sponsored by the authors. No form of support was received from any externa agency for the conduct of this study.

## Competing interests

None disclosed.

## Ethical approval

Not applicable.

## Authors’ contributions

OSI and AAA conceptualized the study. AAA wrote the initial draft of the manuscript. OSI and AAA reviewed the manuscript for critical intellectual content. Both authors approved the final version of the manuscript.

## References

[1] World Health Organization. Coronavirus disease (COVID-19) pandemic. Available from: https://www.who.int/emergencies/diseases/novel-coronavirus-2019?gclid = Cj0KCQjw_dWGBhDAARIsAMcYuJy2TZ2woIcGePAg Rql0eMvHjXjrVGN_wYQ_wdxhCcP4QuA15 RQPM24aAguqEALw_wcB. Accessed June 25, 2021.

[2] Ilesanmi OS, Afolabi AA (2021). Knowledge of community members on COVID-19 in Ibadan, Oyo State, Nigeria. Pan Afr Med J.

[3] Gavi: The Vaccine Alliance. COVAX explained. Available from: https://www.gavi.org/vaccineswork/covax-explained. Accessed December 23, 2020.

[4] Afolabi AA, Ilesanmi OS (2021). Dealing with vaccine hesitancy in Africa: the prospective COVID-19 vaccine context. Pan Afr Med J.

[5] Rief W (2021). Fear of adverse effects and COVID-19 vaccine hesitancy: recommendations of the treatment expectation expert group. JAMA Health Forum.

[6] Solís Arce JS, Warren SS, Meriggi NF, Scacco A, McMurry N, Voors M (2021). COVID-19 vaccine acceptance and hesitancy in low- and middle-income countries. Nat Med.

[7] Ebrahimi OV, Johnson MS, Ebling S, Amundsen OM, Halsøy Ø, Hoffart A (2021). Risk, trust, and flawed assumptions: vaccine hesitancy during the COVID-19 pandemic. Front Public Health.

[8] Dubé E, Laberge C, Guay M, Bramadat P, Roy R, Bettinger J (2013). Vaccine hesitancy: an overview. Hum Vaccin Immunother.

[9] Thunström L, Ashworth M, Finnoff D, Newbold SC (2021). Hesitancy toward a COVID-19 vaccine. Ecohealth.

[10] Ojebode A, Ojebuyi BR, Onyechi NJ, Oladapo O, Oyedele OJ, Fadipe IA. Explaining the Effectiveness of Community-Based Crime Prevention Practices. Ibadan, Nigeria: University of Ibadan; 2016. 10.13140/rg.2.1.1651.9285

[11] Deutsch N, Singh P, Singh V, Curtis R, Siddique AR (2017). Legacy of polio-use of India’s social mobilization network for strengthening of the universal immunization program in India. J Infect Dis.

[12] UNICEF. UNICEF Immunization Roadmap 2018-2030. Available from: https://www.unicef.org/sites/default/files/201901/UNICEF_Immunization_Roadmap_2018.pdf. Accessed June 30, 2021.

[13] Béhanzin L, Adoukonou T, Houeto D, Bokossa C, Agonnoude M (2018). From social determinants of health actions to fight against the Lassa virus hemorrhagic fever epidemic in Tchaourou Commune in Benin, 2018. Open J Epidemiol.

[14] Ilesanmi OS, Akande A, Afolabi AA (2020). Overcoming COVID-19 in West African countries: is herd immunity an option?. Pan Afr Med J.

[15] Folayan MO, Durueke F, Gofwen W, Godo-Odemijie G, Okonkwo C, Nanmak B (2019). Community stakeholder engagement during a vaccine demonstration project in Nigeria: lessons on implementation of the good participatory practice guidelines. Pan Afr Med J.

[16] Bedford J, Chitnis K, Webber N, Dixon P, Limwame K, Elessawi R (2017). Community engagement in Liberia: routine immunization post-Ebola. J Health Commun.

[17] International Association for Public Participation. (2018). IAP2′s public participation spectrum. Available from: https://www.iap2.org.au/Tenant/C0000004/00000001/files/IAP2_Public_Participation_Spectrum.pdf. Accessed July 2, 2021.

[18] Jain M, Engelbert M, Gaarder M, Bagai A, Eyers J (2020). Protocol: use of community participation interventions to improve child immunisation in low‐and middle‐income countries: a systematic review and meta‐analysis. Campbell Syst Rev.

[19] Sentell T, Agner J, Pitt R, Davis J, Guo M, McFarlane E (2020). Considering health literacy, health decision making, and health communication in the social networks of vulnerable new mothers in Hawai’i: a pilot feasibility study. Int J Environ Res Public Health.

[20] Schoch-Spana M, Brunson EK, Long R, Ruth A, Ravi SJ, Trotochaud M (2021). The public’s role in COVID-19 vaccination: human-centered recommendations to enhance pandemic vaccine awareness, access, and acceptance in the United States. Vaccine.

[21] Oyo-Ita A, Wiysonge CS, Oringanje C, Nwachukwu CE, Oduwole O, Meremikwu MM (2016). Interventions for improving coverage of childhood immunisation in low- and middle-income countries. Cochrane Database Syst Rev.

